# Silent Contaminants: Antibiotic‐Resistant Bacteria in Food Systems

**DOI:** 10.1002/mbo3.70418

**Published:** 2026-11-03

**Authors:** Răzvan Odocheanu, Mădălina Lorena Medeleanu, Doru Pamfil, Dan Cristian Vodnar

**Affiliations:** ^1^ Department of Food Science, Faculty of Food Science and Technology University of Agricultural Science and Veterinary Medicine Cluj‐Napoca Cluj‐Napoca Romania; ^2^ Romanian Academy, Cluj–Napoca Branch Cluj‐Napoca Romania

**Keywords:** antimicrobial resistance, antimicrobial resistant bacteria, food systems, one health, resistance genes

## Abstract

Anatimicrobial resistance (AMR) is a rapidly escalating global health crisis, undermining the effectiveness of therapeutic interventions and posing a significant threat to food security, sustainable agriculture, and public health. In 2019, drug‐resistant bacterial infections accounted for an estimated 1.27 million deaths worldwide and are expected to cause 10‐fold more deaths by 2030. Framed within the One Health approach, this review critically examines the role of food systems in the emergence, selection, and dissemination of antimicrobial‐resistant bacteria (ARB) and resistance genes (ARGs) across animal, plant, and aquatic sectors. Through bibliometric network analysis (2015–2025), we highlight research trends and thematic clusters that shape current understanding of AMR in the food chain. Key pathways include antibiotic use in livestock and aquaculture, contamination of crops via irrigation and fertilization, and post‐harvest practices that sustain ARB persistence. Mechanistic insights into AMR are discussed, including enzymatic degradation (e.g., blaTEM), target modification, efflux pumps, and mobile genetic elements that mediate horizontal gene transfer. The review also evaluates control strategies, regulatory frameworks (e.g., EU legislation on AMR monitoring), integrated surveillance systems (e.g., EFSA–ECDC reports, WHO GLASS), and cross‐sectoral approaches such as One Health. Emphasis is placed on public health interventions that limit exposure and transmission of antimicrobial‐resistant pathogens across the food chain, underscoring the need for coordinated, multisectoral action that positions food safety as a core pillar of global health resilience.

## Graphical abstract

This graphical abstract illustrates the “farm‐to‐fork” cycle of antibiotic resistance, highlighting how antibiotic use promotes resistant bacteria that spread through animals, crops, food, and humans, emphasizing the interconnected nature of antimicrobial resistance and the need for integrated One Health strategies to mitigate its global impact.

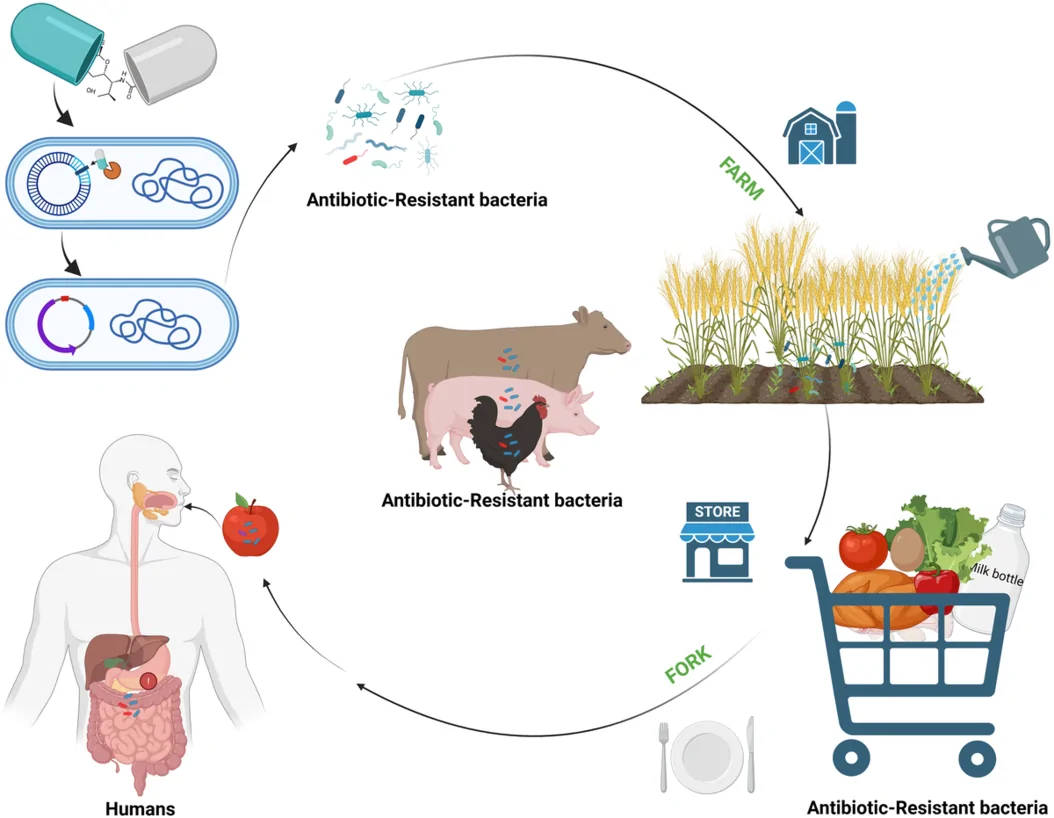

## 1 Introduction

Antimicrobial resistance (AMR) spreads across humans, animals, plants, and the environment, reducing the effectiveness of treatments and threatening health, food security, and livelihoods. Antimicrobial agents, notably antibiotics, constitute a cornerstone of modern therapeutic interventions, being indispensable for the prophylaxis and treatment of bacterial infections in humans and animals, as well as for the control of phytopathogens in agricultural crop production. AMR arises when microorganisms undergo changes that render them unaffected by antimicrobial agents. AMR is acknowledged by the World Health Organization (WHO) as one of the most significant global health challenges of the 21st century (Coque et al. 2023; Walsh et al. 2023).

In 2019, AMR was linked to 1.27 million deaths and is considered a top global health threat. In 2021, AMR was estimated to be associated with 4.71 million deaths globally (95% uncertainty interval [UI]: 4.23–5.19 million), of which 1.14 million (95% UI: 1.00–1.28 million) were directly attributable to resistant bacterial infections (Naghavi et al. 2024). Mortality trends linked to AMR shifted considerably across age groups and regions between 1990 and 2021: while deaths in children under five fell by more than half over this period, mortality among adults aged 70 and older rose by over 80%, highlighting a marked age‐related redistribution of the AMR burden (Murray et al. 2022; Naghavi et al. 2024). By 2050, AMR is projected to cause up to 10 million deaths annually (Tang et al. 2023), while its global economic impact is expected to reach $100 trillion USD (O'Neill 2014). By contrast, while AMR‐related deaths are projected to rise sharply by 69.6% (95% UI: 51.5%–89.2%) between 2022 and 2050, the corresponding increase in disability‐adjusted life years (DALYs) is expected to be far more modest, at just 9.4% (95% UI: –6.9% to 29.0%), reaching an estimated 46.5 million DALYs (95% UI: 37.7–57.3 million) by 2050 (Naghavi et al. 2024). Although AMR arises naturally, its development has been significantly accelerated by the inappropriate use of antimicrobials, including overuse, underuse, and misuse, in both human and veterinary contexts (Dhingra et al. 2020; Founou et al. 2021). From the beginning, antibiotics aimed to reduce and, ideally, eliminate the growth of microorganisms.

In addition to their primary use in human medicine, antibiotics are widely employed in agriculture to boost crop yields, in animal husbandry for treating infections, in prophylactic and metaphylactic applications to prevent disease, and as growth promoters in animal feed at regulated concentrations. Bacteria, on the other hand, have developed multiple mechanisms to acquire resistance to these compounds (EFSA 2021; Mann et al. 2021; Salam et al. 2023).

In the face of the increasing AMR threat, global health organizations and governments have initiated joint responses. The *One Health* initiative was implemented to facilitate interdisciplinary work among organizations such as the Food and Agriculture Organization (FAO) and the World Organization for Animal Health (WOAH), and to harmonize actions across sectors. Finally, the WHO announced the *Global Action Plan* on AMR (GAP‐AMR) and created the *Global Antimicrobial Resistance* and *Use Surveillance System* (GLASS), respectively, to ensure that knowledge gaps can be addressed and support the implementation of the GAP‐AMR and its goals (de Mesquita Souza Saraiva et al. 2022; Sweileh 2021; Tang et al. 2023).

The One Health framework focuses on evaluating the risks linked to the emergence, spread, and sustainability of AMR within the interconnected realms of human, animal, and environmental health. These areas comprise diverse populations that interact to varying degrees and possess different capacities to influence AMR trends. Hence, One Health concepts directly influence AMR and its evolution in different industries (Allel et al. 2023; Coque et al. 2023). For example, using avoparcin as a growth promoter in livestock was associated with the rise of vancomycin‐resistant enterococci (VRE) in both animals and humans. Its ban in the European Union was then followed by a significant decline in VRE prevalence in both groups (G. Li et al. 2023). Similarly, the widespread use of colistin in pig farming, especially in China, contributed to the emergence and global spread of the mobile resistance gene mcr‐1, leading to its ban as a feed additive in 2017 (Touati et al. 2025). In environmental contexts, effluents from pharmaceutical manufacturing in India have been found to create antibiotic‐rich water bodies that serve as reservoirs for multidrug‐resistant (MDR) bacteria (Kotwani et al. 2021; 2022; 2023). Antimicrobials are widely used in food‐producing animals, potentially serving as a reservoir for antibiotic‐resistant bacteria that can be transmitted to humans through the food chain, direct contact, or the environment (Milijasevic et al. 2024; Nastasijevic et al. 2024). They are administered mainly on four occasions: treating diseased animals, metaphylaxis of groups with some infected animals, prophylaxis of healthy animals at risk, and growth promotion with sub‐therapeutic doses. The last three uses are most closely associated with resistance development, which makes surveillance along meat and aquaculture chains a key part of One Health monitoring (Nastasijevic et al. 2024; Nulty et al. 2016). Regulatory frameworks vary significantly: in the EU, Regulation (EU) 2019/6 and Regulation (EU) 2019/4, effective since January 2022, prohibit growth promotion and routine group prophylaxis, limit metaphylaxis to justified high‐risk cases, and extend these bans to imported animal products (Article 118), leading to about a 50% decrease in veterinary antimicrobial sales over the past decade. In the United States, similar objectives were achieved more gradually, with the Food and Drug Administration (FDA) issuing Guidance for Industry that banned growth‐promoting uses while still allowing disease prevention in healthy animals, a practice the WHO recommends discontinuing (European Medicines 2024; Food and Drug 2024; WHO 2017). Within this context, food is considered a key vector that can facilitate the entry of resistant pathogens into the food chain (Ferreira et al. 2021).

Bacteria harboring ARGs in animals can reach humans directly through the food chain *via* the consumption of undercooked or raw food, handling of contaminated products, or cross‐contamination with other foods, or indirectly through environmental pathways. In addition, direct transmission can occur through contact with infected animals on farms (Bennani et al. 2020). EFSA documented the transmission of zoonotic bacteria such as *Campylobacter, Salmonella,* and *Escherichia coli*, via mechanisms including water or environmental contamination and direct contact with animals (EFSA 2022). Since AMR depends on the presence of live microorganisms and the transfer of ARGs, any food processing step that alters the bacterial load can significantly influence the risk of exposure to AMR. For this reason, food categories are considered based on their potential to support or inhibit bacterial survival and growth. While specific effects may vary by microorganism, general trends in microbial behavior can be identified across food types (Ahmed et al. 2024).

EFSA categorizes key food categories relevant to the transmission of antimicrobial‐resistant bacteria, including products of animal origin (e.g., fish, beef meat), plant‐based foods, and water used directly in the food chain. In addition, mixed‐origin products, such as ready‐to‐eat and convenience foods that often combine ingredients from multiple sources, represent a distinct and significant category in AMR exposure pathways (EFSA 2008). Effectively controlling all pathways through which AMR bacteria and their associated genes can reach humans, including, but not limited to, the food chain, requires a coordinated response from all stakeholders. Each sector, from human and veterinary medicine to food animal production, processing, preparation, and food safety regulation, must take responsibility for preventing both the emergence and dissemination of AMR within its respective domains. Healthcare providers in human and veterinary fields must prescribe antibiotics responsibly, only when necessary, and avoid those critical to human health. Regulatory agencies are tasked with authorizing, controlling, and monitoring veterinary antimicrobials, including phasing out their use for growth promotion. Producers should promote good husbandry, biosecurity, and vaccination to minimize the need for treatment. Food businesses need to maintain hygiene and follow HACCP protocols to limit the spread of resistant bacteria during processing. Food safety authorities are responsible for establishing microbiological standards and surveillance of AMR throughout the supply chain. Tackling the emergence and spread of AMR depends on each sector performing its role within a unified, cross‐sector strategy (Codex Alimentarius 2021).

This work points out the challenges of AMR in food systems, considering the occurrence of resistant bacteria in different food matrices and the mechanisms involved in their persistence and distribution. Furthermore, the health effects in humans from consuming products contaminated with resistant bacteria or genes, the pathways leading to such contamination, as well as the mechanisms involved in the development of these genes, were addressed. Addressing AMR requires integrated, multisectoral measures aligned with the One Health approach. While regulations to reduce antimicrobial use have been adopted globally, this study offers updated insights into high‐risk AMR pathogens in livestock by synthesizing recent international research. It further examines the spread of resistance genes, their impact on animal and human health, and outlines strategic approaches to mitigate this global threat.

A bibliometric co‐occurrence network was generated in VOSviewer (Figure 1) from author keywords of articles indexed in Scopus between 2015 and 2025 using the search terms “food,” “antimicrobial resistance,” and “multidrug resistance,” with a minimum threshold of 10 occurrences per keyword. Central to the map is “antimicrobial resistance,” closely linked with “multidrug resistance,” “antibiotic resistance,” and “one health,” reflecting a concerted focus on resistance phenotyping within an integrated human‐animal‐environment framework. A primary cluster highlights zoonotic bacteria, most notably *Salmonella enterica* and *Campylobacter* spp., alongside “plasmids,” “resistance genes,” and “virulence,” emphasizing pathogen surveillance and horizontal gene transfer. The genomics cluster (blue), including “whole‐genome sequencing,” “MLST,” and “genomics,” underscores high‐resolution tools for tracing transmission and source attribution across the food chain. The biofilm‐focused module (red) interconnects “biofilm,” “antimicrobials,” and “antibiotics,” drawing attention to the persistence of resistant populations on food contact surfaces and in processing environments, where sessile communities exhibit enhanced tolerance to sanitizers and therapeutic agents. This cluster converges with nodes representing “Staphylococcus aureus,” “Bacillus cereus,” and “Enterobacteriaceae,” indicating the relevance of both Gram‐positive and Gram‐negative taxa in biofilm‐associated resistance. Interdisciplinary linkages emerge in clusters addressing “food security,” “food chain,” and “public health”, reaffirming that antimicrobial use in agriculture permeates food systems, with reciprocal impacts on human and environmental microbial ecology.

**Figure 1:**
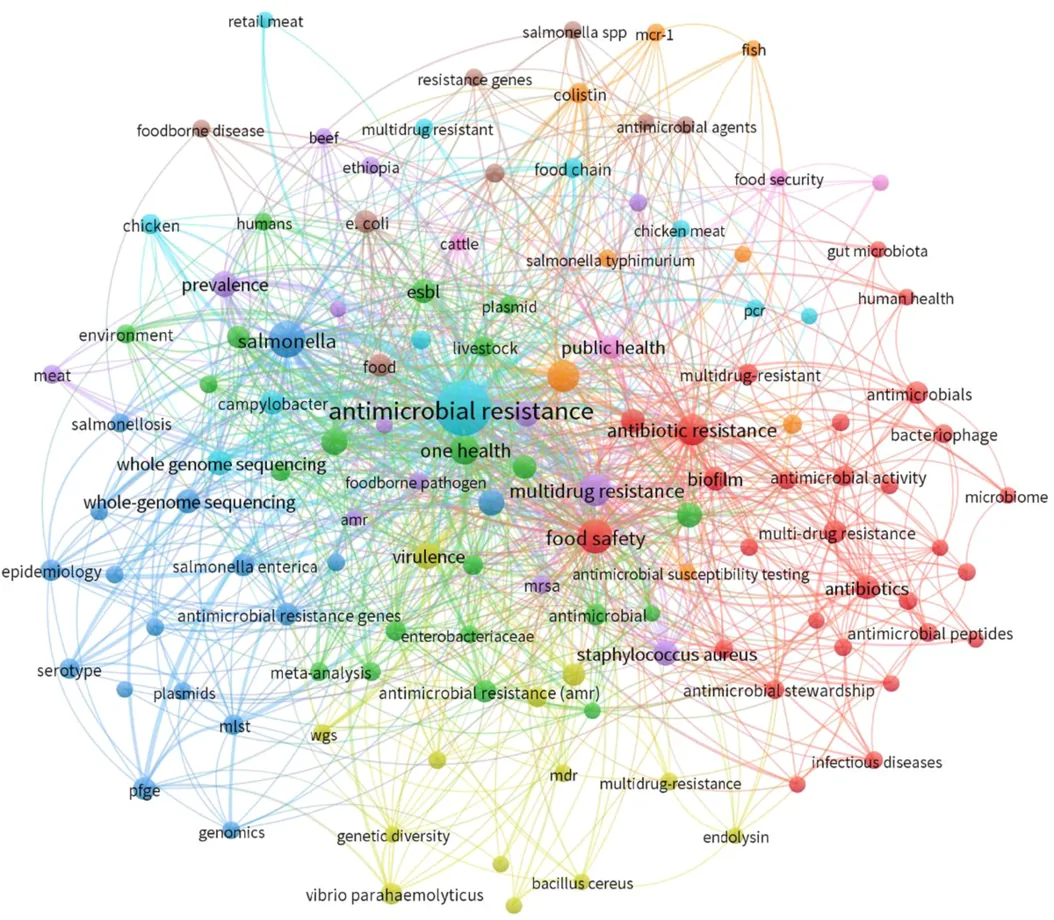
Bibliometric co‐occurrence network of antimicrobial resistance–related keywords in food systems (2015–2025), generated using VOSviewer based on data retrieved from the Scopus database. Node size represents keyword frequency, while link strength indicates co‐occurrence relationships among terms.

## 2 Sources of Antibiotic‐Resistant Bacteria in Food

Over time, resistant bacteria have been responsible for several outbreaks of foodborne illnesses. *Salmonella* infections can affect individuals of all ages, with common foodborne sources including RTE products, eggs, pork, and poultry. *Listeria monocytogenes* primarily poses a risk to the elderly, immunocompromised individuals, and pregnant women, and is associated with high mortality rates; typical sources include RTE foods such as smoked fish and soft cheeses. AMR *E. coli* (Shiga toxin‐producer) can lead to severe complications such as acute kidney failure, particularly in young children, the elderly, and immunocompromised individuals, and is associated with the consumption of meat, vegetables, and unpasteurized juices (Vilne et al. 2019). EFSA (2024) report presents campylobacteriosis and salmonellosis as the most reported zoonoses in humans in 2023, in 27 member states, the United Kingdom, and 10 non‐member states. Surprisingly, *Salmonella* samples from the carcasses of various animal species and *Campylobacter* quantification samples from broiler carcasses showed higher positivity rates and several AMG when tested by competent authorities, compared to results from own‐checks conducted by food business operators. *Salmonella* in eggs and egg products represented the most concerning agent–food combination in 2023, accounting for the highest number of outbreaks and reported cases across the EU. This pairing also ranked second in terms of hospitalizations and was the leading cause of multi‐country outbreaks reported that year (EFSA 2024). Punchihewage‐Don et al. (2022) summarize in their review *Salmonella*‐related outbreaks linked to live poultry, backyard poultry, and raw or undercooked poultry products in the United States. These outbreaks originated from diverse *Salmonella* serotypes, including *S. enteritidis, S. infantis, S. hadar, and S. typhimurium*, many of which exhibited resistance to multiple antibiotic classes. Frequently reported resistance to ampicillin, tetracycline, streptomycin, gentamicin, chloramphenicol, ciprofloxacin, and trimethoprim‐sulfamethoxazole, suggesting a growing prevalence of MDR strains. Resistance was often traced to genetic elements within strains associated with poultry, especially those raised in backyard settings, where antibiotic use and biosecurity measures are poorly regulated. The high number of cases—for example, 1135 in a 2021 backyard poultry outbreak—illustrates the public health risk posed by zoonotic transmission from food animals to humans, exacerbated by the persistence and mobility of AMR determinants (Punchihewage‐Don et al. 2022). Household‐prepared foods are associated with more cases of foodborne illness than foods consumed elsewhere. Food‐handling behaviors in private kitchens play a key role in transmitting AMR bacteria. Implementing basic hygiene measures effectively prevents contamination with these bacteria. The main routes of bacterial spread in the kitchen involve inadequate washing of contaminated hands, knives, and cutting boards during and after food preparation (Freivogel & Visschers 2020).

### 2.1 AMR in the Meat, Poultry, Dairy, and Egg Industry

Antibiotics are widely used in agriculture, livestock, poultry, fisheries, and animal husbandry, not only therapeutically to treat diagnosed infections but also for metaphylaxis and prophylaxis, and, in some regions, for growth promotion. Among the approximately 30 antibiotics commonly used in these sectors, macrolides, penicillins, and tetracyclines are the most prevalent classes (Mann et al. 2021). Lately, antibiotics have expanded beyond their therapeutic applicability in animal sectors, being used as growth promoters to enhance the production of meat, dairy, and egg products. This problem is particularly prevalent in underdeveloped countries where the population exceeds a manageable size. For this reason, the growing demand required an increase in the production yields for animal‐source foods, which led farmers and people involved in the farm‐to‐fork chain to use antibiotics in animal diets (such as poultry, chicken, and cattle) as growth promoters to meet the public demand (M. M. Hassan et al. 2021). According to Adegbeye et al. (2024), approximately 9 million kilograms of antibiotics are administered annually worldwide to treat infections, while around 3 million kilograms are applied for prophylactic purposes or to promote growth (Adegbeye et al. 2024). Unfortunately, their excessive and inappropriate use was not well documented, thus facilitating the emergence of resistant bacteria in the human body through the food chain (the presence of antibiotic residues in animal organs, body tissue, or milk) or environmental spreading (30%–90% of veterinary antibiotics are transferred to the marine environment via urine, feces, and wastewater) (Figure 2) (Redwan Haque et al. 2023). International trade accelerates this process, enabling resistant bacteria from regions with weaker regulations to spread from developing to developed nations. A well‐known case is the MDR *S. enterica* serotype Kentucky ST198, which is highly resistant to ciprofloxacin. It was first identified in Egypt in the early 2000s and later traveled across Africa, the Middle East, Europe, and North America through the poultry trade and travel (Le Hello et al. 2011). Recently, the mcr‐3 and mcr‐9 genes, responsible for transmissible colistin resistance, were found in bacteria from seafood imported into the United States, which sources 65%–85% of its seafood from abroad, but not in domestically produced seafood. This highlights how aquaculture from regions with limited antimicrobial oversight can introduce resistance genes of last resort into importing countries (J. W. Hassan et al. 2025). The selective accumulation of antibiotic residues in food products is closely linked to their chemical class and physicochemical behavior. Lipophilic antibiotics, such as tetracyclines and macrolides, tend to associate with fat and protein fractions, leading to higher concentrations in dairy matrices like curd, cheese, and cream (Gajda et al. 2017; Giraldo et al. 2017). In contrast, hydrophilic compounds like β‐lactams are more water‐soluble and are predominantly found in whey or expelled during processing (Giraldo et al. 2017). These patterns are driven by factors such as protein binding affinity, fat solubility, and tissue‐specific metabolism (Adegbeye et al. 2024). For example, H. F. Hassan et al. (2021) revealed in their study a consistent tendency for antibiotics to preferentially accumulate in visceral organs rather than in muscle tissue, likely due to organ‐specific metabolic activity and the chemical affinity of antibiotics based on their lipophilic or hydrophilic properties. Higher concentrations of oxytetracycline, tetracycline, amoxicillin, and tylosin were accumulated in the kidneys of sheep compared to the liver and muscle (H. F. Hassan et al. 2021; Sallam et al. 2022). Similarly, a higher prevalence of antibiotics was reported in chicken giblets than in chicken meat and eggs (Yang et al. 2020).

**Figure 2:**
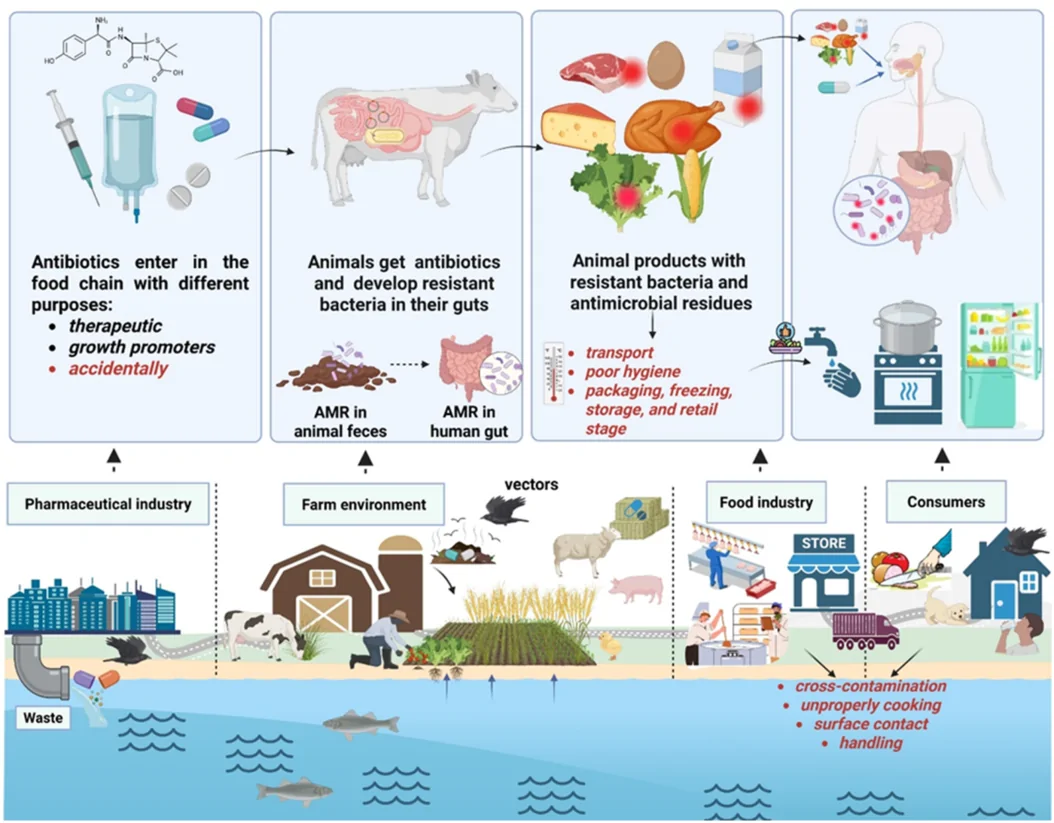
Schematic representation of antimicrobial resistance (AMR) flow in the food system—from farm to consumer. The diagram illustrates the One Health interconnection between animal, environmental, and human health.

Literature focuses on chicken, cattle, and pigs as the most frequently used animal sources for AMR hazards (Fastl et al. 2023). Aquaculture is a crucial yet frequently underrecognized source: antimicrobials are often added to feed for whole production units and remain at sub‐therapeutic levels in water and sediments. This leads to the selection and spread of resistant bacteria and their genes in open aquatic systems, ultimately reaching humans through the consumption of fish and seafood (Milijasevic et al. 2024). Resistant bacteria, such as *Salmonella* spp., *Campylobacter* spp., *Staphylococcus* spp., *L. monocytogenes*, and *E. coli*, are prevalent in animals and can be transmitted to humans via the food chain, resulting in health concerns. *Salmonella* is the primary resistant bacterium found in poultry meat and eggs, with a high likelihood of transmission to humans, which can potentially lead to salmonellosis. Redwan Haque et al. (2023) noted the resistance of *S. enterica* isolated from eggs and chicken carcasses against penicillin, streptomycin, tetracycline, quinolone, and sulfisoxazole, which can then infiltrate people's diet, causing gastrointestinal diseases (Redwan Haque et al. 2023). For example, Sarker et al. (2021) reported the detection of *S. enterica* in 40% of layer chickens. Furthermore, they observed that the isolated strains exhibited resistance to penicillin (100%), nalidixic acid (100%), sulfamethoxazole‐trimethoprim (55%), ampicillin (40%), and amoxicillin (25%) (Sarker et al. 2021). Similarly, Alam et al. (2020) have found *S. enterica* serovar *Typhimurium* in 30% of broiler chickens tested. The isolates registered the highest resistance against tetracycline (97%) and chloramphenicol (94%), followed by ampicillin (83%) and streptomycin (77%) (Alam et al. 2020). These findings highlight a widespread pattern of multidrug resistance (MDR), resistance to at least three classes of antimicrobials, in non‐typhoidal Salmonella (NTS), a major foodborne pathogen that causes approximately 94 million infections and over 150,000 deaths each year globally (Stanaway et al. 2019). MDR‐NTS is particularly concerning because resistance to frontline drugs like fluoroquinolones and third‐generation cephalosporins hampers the treatment of serious infections. This is why the WHO identified fluoroquinolone‐resistant NTS as a high‐priority pathogen in its 2024 Bacterial Priority Pathogens List (WHO 2024c). The widespread overuse and misuse of antimicrobials in egg and other animal‐source food chains are primarily driven by economic and regulatory factors. High stocking densities in intensive layer and broiler systems increase disease risk, leading to prophylactic use to maintain productivity. In addition, the easy availability of antimicrobials over the counter, weak enforcement of regulations, and limited veterinary services, particularly in low‐ and middle‐income countries, permit these drugs to be routinely used in healthy animals without proper diagnostic confirmation (Ehsan 2025).

Food production serves as a pathway for the transmission of both antibiotic ARGs and antibiotic‐resistant bacteria to humans (Asghar et al. 2024). For instance, Cao et al. (2022) analyzed over 1000 gut metagenomes from humans, pigs, and chickens, revealing a significant overlap of antimicrobial ARGs among these hosts. They identified shared ARGs conferring resistance to multiple antibiotic classes, including aminoglycosides, tetracyclines, and beta‐lactams, with mobile genetic elements such as transposons Tn4451/Tn4453 and TnAs3 facilitating their transfer. Beyene et al. (2017) carried out a study to assess the presence of microorganisms in 193 samples (milk, meat, equipment, and humans) from dairy farms and abattoirs. A higher prevalence was observed for *Staphylococcus* spp. in dairy farms (50%) compared to abattoirs (47.7%). The results also revealed that the strains exhibited resistance to penicillin‐G (95,3%), nalidixic acid (88.4%), cloxacillin (79.1%), vancomycin (65.1%), and cefoxitin (55.8%) (Beyene et al. 2017). Aquaculture is a similar path. Fukuda et al. (2023) analyzed 85 retail raw seafood samples in Japan, finding mainly Vibrio spp. (54.1%) and Aeromonas spp. (34.1%). They detected isolates resistant to tetracycline, sulfamethoxazole, and trimethoprim/sulfamethoxazole that carried tet, sul, and dfr genes in 21.9% of the fish samples. Resistance to sulfamethoxazole was found only in farm‐raised fish, directly linking this resistance pattern to the use of antimicrobials in aquaculture (Fukuda et al. 2023). A study from Almansour et al. (2023) describes *L. monocytogenes* as a cause of listeriosis, a severe infection that can lead to septicemia, meningitis, and other complications, primarily affecting ruminants but also other animal species and humans. It accounts for up to 24% of foodborne illness‐related deaths, posing significant public health and economic burdens. Certain strains have shown resistance to antibiotics such as tetracyclines, erythromycin, and ciprofloxacin (Almansour et al. 2023).

### 2.2 Plant‐Source Foods: Contamination via Manure, Irrigation, Handling

Although antibiotic residues in food have traditionally been associated with their use in livestock for therapeutic or growth‐promoting purposes, accumulating evidence suggests that plant‐derived foods may also serve as important reservoirs of AMR. Specific environmental matrices, including those adjacent to pharmaceutical manufacturing sites, intensive livestock operations, aquaculture systems, and wastewater treatment facilities receiving effluents from healthcare institutions, are persistently exposed to antibiotic‐laden contaminants. This continuous environmental loading contributes to the prolonged presence and selective pressure of sub‐therapeutic to therapeutic concentrations of antimicrobials, thereby facilitating the emergence and dissemination of resistance determinants (Serwecińska 2020). Engaging directly with animals or their natural habitats serves as a significant supplementary avenue, particularly for individuals involved in agriculture or animal husbandry. In such environments, humans may have close interactions with animals or inhale dust particles that can disseminate resistant bacteria (Ifedinezi et al. 2024).

Foods of plant origin can become contaminated with AMR bacteria from diverse environmental sources. Soil, a natural reservoir of bacteria harboring AMR genes, poses a significant risk when edible plant tissues come into direct contact with it or are exposed to soil splashes (Zeng et al. 2025; Y.‐J. Zhang et al. 2019). Plants absorb antibiotics with varying distribution patterns within their tissues. Tetracyclines, fluoroquinolones, and chloramphenicol tend to accumulate more in fruits than in leaves or roots, whereas sulfonamides and macrolides preferentially localize in roots over aerial parts (Pan and Chu 2017). The application of animal or human waste as soil amendments, as well as contamination from animal intrusion, further increases the likelihood of AMR bacterial transfer. In addition, irrigation water may serve as a vector for AMR organisms, facilitating their dissemination onto crops (Group 2018). This is especially important when treated municipal wastewater (reclaimed water), surface water from rivers receiving effluents, or untreated wastewater is used for irrigation, a practice now implemented on over 20 million hectares across more than 50 water‐scarce countries (Gomes et al. 2026). Since conventional treatments do not entirely eliminate antibiotic residues, resistant bacteria, and resistance genes, these contaminants can settle on crop surfaces, transfer to native soil bacteria via horizontal gene transfer, and eventually be absorbed by edible plants, reaching consumers (Slobodiuk et al. 2021). An extensive review encompassing over 100 studies conducted over the past 40 years on the presence of pharmaceutical compounds in animal waste has highlighted the widespread occurrence of several antibiotics, with enrofloxacin, oxytetracycline, and chlortetracycline being reported at the highest concentrations. These antimicrobials were consistently detected in both raw and processed manure derived from various livestock production systems (Ghirardini et al. 2020).

The application of manure as an organic fertilizer is a recognized driver of ARG dissemination within crop‐based agricultural systems, thereby posing potential public health risks. The high organic matter content in manure promotes the proliferation of both intrinsic and extrinsic ARB. Studies have shown that ARGs such as *aadE*, *sat4*, *tetX*, *dfrA1*, *floR*, *ermF*, and *aadA* are frequently detected in poultry manure compared to swine or cattle manure, with elevated concentrations of tetracyclines, sulfonamides, and macrolides also commonly observed (Osman et al. 2018). Moreover, organically grown lettuce has been reported to exhibit higher ARG abundance and diversity than conventionally grown lettuce. For example, in Chinese cabbage (*Brassica pekinensis*), increased ARG carriage within the leaf endosphere was linked to manure application (Y. Zhang et al. 2023). Preventing manure‐associated AMR depends on treating manure prior to land application instead of spreading it raw. Composting and anaerobic digestion are the primary methods, which decrease the abundance of antibiotic resistance genes (ARGs) mainly due to the high temperatures during their thermophilic stages. These temperatures kill bacteria that carry ARGs and inhibit horizontal gene transfer. For instance, thermophilic anaerobic digestion of dairy manure at 52°C has achieved reductions of up to 95% in sul2 and int1 (Wang et al. 2021). However, treatment does not completely eliminate resistance, as genes such as sul1 and sul2 can persist or be re‐enriched during maturation. This highlights the importance of optimizing treatment parameters (Zhao et al. 2025). In addition, evidence of plasmid‐mediated horizontal gene transfer from bacteria isolated from fresh produce (e.g., vegetable salads, arugula, and cilantro) to *E. coli* suggests that plant‐associated microbiota may serve as reservoirs for ARGs, facilitating their transfer to human gut microbes upon consumption. The high prevalence and diversity of ARGs have been predominantly associated with bacterial taxa belonging to the phyla *Proteobacteria*, *Firmicutes*, *Bacteroidetes*, and *Actinobacteria*. Among these, *Actinobacteria* are considered major contributors to soil‐borne ARGs, while *Proteobacteria* are notable for carrying broad‐host‐range plasmids that facilitate horizontal gene transfer. Plants serve as reservoirs for a wide array of endophytic bacteria, which establish complex interactions within plant tissues (Tripathi et al. 2024). Municipal wastewater treatment plants also serve as significant reservoirs for antibiotics and their transformation products. Despite conventional treatment processes, many of these compounds are not eliminated and persist in the effluent. As treated municipal wastewater is released into environmental compartments such as surface waters, groundwater, and agricultural soils, it introduces sublethal concentrations of antibiotics that can exert selective pressure on microbial communities (Phan et al. 2024). For example, Rodriguez‐Mozaz et al. (2020) detected 53 antibiotics in final effluents from wastewater treatment plants in seven European countries (Portugal, Spain, Ireland, Cyprus, Germany, Finland, and Norway). The study quantified a range of antibiotic classes, including penicillins (26–48 ng/L), cephalosporins (25–39 ng/L), tetracyclines (32–38 ng/L), sulfonamides (21–37 ng/L), and nitroimidazoles (17–41 ng/L), indicating widespread and consistent contamination (Rodriguez‐Mozaz et al. 2020). The consumption of various fresh leafy and non‐leafy vegetables, root vegetables, sprouts, and fruits exposes humans to foodborne bacterial pathogens, including antibiotic‐resistant strains (Rahman et al. 2022). Literature reports *E. coli* as the primary source of contamination for fresh produce, followed by *Salmonella* spp. For example, Shah et al. (2015) evaluated 260 vegetable and salad mix (cucumber, lettuce, spinach, and carrot) samples from the markets for the identification of pathogenic resistant bacteria. The results show a contamination of 34% of vegetables and 26.7% of salad samples with different *E. coli* pathotypes. Nonetheless, a total of 92% of diarrheagenic *E. coli* pathotypes exhibited resistance to tetracycline, while 87% were resistant to ampicillin (Shah et al. 2015). In the United States, 100,000 fresh produce samples were analyzed, revealing *Salmonella*spp. in 152 samples across 51 serotypes. The most significant antibiotic‐resistant serotypes were detected on lettuce (23.1%) and cantaloupe (6.7%) (Reddy et al. 2016).

This fosters the emergence and propagation of AMB and ARGs in both aquatic and terrestrial ecosystems, highlighting the critical need for integrated One Health approaches to monitor and mitigate AMR across environmental, agricultural, and human health sectors.

### 2.3 AMR in Aquaculture and Fisheries

Aquaculture and fisheries are increasingly significant contributors to global nutritional needs, currently accounting for approximately 20% of the animal‐derived protein intake for over 40% of the global population, with consumption rates rising faster than those of any other animal protein source (FAO 2020). However, the aquatic food production sector may represent an under‐recognized pathway for the transmission of antibiotic‐resistant bacteria (ARB) and ARGs. This transmission can occur through direct or indirect routes, involving aquatic animals, their surrounding environments, and human populations. Schar et al. (2021) conducted a predictive assessment of AMR in marine environments across Asia, identifying several coastal regions with particularly high levels of MDR bacteria.

The highest predicted AMR prevalence was observed in the northeastern coastal waters of China, including the Yellow Sea and East China Sea; the eastern coastal waters of Hainan Island in southern China; the central coastal areas of Vietnam along the South China Sea; the coastal waters of southern India, including the Arabian Sea and the Bay of Bengal between southern India and northern Sri Lanka; and the eastern Mediterranean coast of Lebanon (Schar et al. 2021). Among the 11 Southeast Asian countries, Vietnam, Malaysia, and Thailand reported the highest number of AMR cases, as documented by Suyamud et al. (2024). Resistance was observed across 17 antibiotic classes, with beta‐lactams, tetracyclines, sulfonamides, and aminoglycosides being the most frequently detected.

Predominant ARB included *E. coli*, *Aeromonas* spp., and *Vibrio* spp., which exhibited multiple antibiotic resistance indices exceeding 0.2, surpassing the global average and indicating significant selective pressure (Suyamud et al. 2024). Notably, ARGs such as *blaTEM*, *blaSHV*, *sul2*, and *dfrA* were widely detected within aquatic ecosystems, suggesting active horizontal gene transfer mechanisms facilitating the dissemination of AMR (Nguyen et al. 2014). Contributing factors include the overuse and misuse of antimicrobials in aquaculture, suboptimal farm management practices, and environmental variables such as elevated temperatures and degraded water quality. In Vietnam and Thailand, several MDR genes have been identified, including *ermB*, *norM*, *floR*, *adeF*, and *aac(6′)‐Ib‐cr*. These genes confer resistance through distinct molecular mechanisms: *ermB* mediates antibiotic target modification by methylating 23S rRNA, leading to resistance against macrolides (Dzyubak and Yap 2016); *norM*, *floR*, and *adeF* are associated with active antibiotic efflux, reducing intracellular drug concentrations; while *aac(6′)‐Ib‐cr* encodes an aminoglycoside acetyltransferase that inactivates antibiotics through acetylation (Gharbi et al. 2023).

Probiotics have emerged as effective alternatives to antibiotics in aquaculture, contributing to enhanced health and disease resistance in farmed species. A variety of commercial probiotic formulations are currently available for use. For instance, the administration of probiotics to *Litopenaeus vannamei* (Pacific white shrimp) significantly enhanced immune responses, as evidenced by increased phagocytic activity and resistance to *Vibrio parahaemolyticus* infections (Lee et al. 2022). In addition, the application of medicinal herbs, such as onion, ginger, and olive, has demonstrated a wide range of bioactive properties, including antiviral, antibacterial, and antifungal effects. These botanicals also offer physiological benefits, including hormonal regulation and immune and digestive system support, in a variety of economically important aquaculture species such as marine fish (e.g., rainbow trout and sea bream), freshwater fish (e.g., carp), and crustaceans (e.g., shrimp) (M. Li et al. 2022).

### 2.4 Post‐Harvest Contamination in the Supply Chain

Ensuring the quality and microbiological safety of fruits and vegetables from harvest to retail is critical to the produce industry, as it guarantees the delivery of abundant and nutritionally valuable food to consumers. Fresh produce is susceptible to microbial contamination at various stages of the supply chain, including cultivation, harvesting, and postharvest handling, with the risk being influenced by a range of environmental and operational factors (Dallaire et al. 2006). Numerous studies have identified ARB and ARGs on fresh produce. In Japan, extended‐spectrum β‐lactamase‐producing pathogens have been detected on vegetables. MDR *E. coli* and *Salmonella* spp. have been implicated in several outbreaks across Germany, the USA, Canada, Australia, and Finland. In addition, opportunistic pathogens such as *Klebsiella* spp. and *Enterobacter* spp. have been isolated from retail vegetables (e.g., cabbage, tomatoes), posing a risk of severe infections, particularly in immunocompromised individuals (Rahman et al. 2021).

Primary sources include direct contact with fecal matter during cultivation, such as through wastewater irrigation, biosolids, or animal manure application (Chee‐Sanford et al. 2009; Jung et al. 2014). Improper post‐harvest handling practices, such as cutting, washing, packaging, and storage, can facilitate microbial cross‐contamination of fresh produce. Contaminated wash water, reuse of wash solutions, and residual soil particles are notable sources, including contamination with pathogens like *Arcobacter* spp. In addition, poor hygiene among food handlers, particularly in retail markets, contributes to contamination risks. To mitigate these ARB issues, studies recommend: (i) enforcing hygiene and sanitation protocols across the supply chain; (ii) regulating and monitoring the use of water for washing and sprinkling; (iii) adopting non‐chlorine sanitizers during post‐harvest treatment (peracetic acid, ozonated water, hydrogen peroxide, and organic acids) (Jin & Adhikari 1924; Rico et al. 2007); and (iv) improving vendor hygiene and cleanliness of holding containers regardless of market setting (Gómez‐Aldapa et al. 2016; He et al. 2020; Rahman et al. 2021).

## 3 Mechanisms and Persistence of AMR in the Food Chain

### 3.1 Bacteria Involved in AMR Persistence in the Food Chain

In the context of the One Health approach, numerous studies have focused on key bacterial pathogens capable of acquiring AMR mechanisms, particularly *Enterococcus faecium*, *S. aureus*, *Klebsiella pneumoniae*, *Acinetobacter baumannii*, *Pseudomonas aeruginosa*, *Shigella* spp., *Enterobacter* spp., and *E. coli* (Despotovic et al. 2023). These microorganisms are also included in the WHO list of priority pathogens due to their critical clinical relevance, being frequently associated with resistance to last‐resort antimicrobial agents (WHO 2024b). Many of these last‐resort agents are listed in the WHO List of Medically Important Antimicrobials (MIA List), a tool that prioritizes antimicrobial classes based on their importance for human health, ranging from “important” to “highest priority critically important antimicrobials” (HPCIA). The HPCIA category includes third‐ and fourth‐generation cephalosporins, quinolones, and polymyxins like colistin. By highlighting agents that require their efficacy to be conserved, the list seeks to limit their non‐human use across human, animal, and environmental sectors, aligning with a One Health approach (WHO 2024d). Their occurrence has been extensively documented across multiple niches, including human, animal, and environmental reservoirs.

Specifically, vancomycin‐resistant *Enterococcus faecium* (VREfm) represents a critical example of this phenomenon. Historically, livestock was implicated as a potential reservoir for VREfm and vancomycin ARGs, linked to the use of avoparcin as a growth promoter in Europe until its ban in 1997 (WHO 2024b). Subsequent studies reported a rising incidence of VREfm in broilers, particularly involving sequence types ST10, ST13, and ST370. However, differentiating strains derived from food sources versus animal origins remains challenging. Within the framework of “exclusive mutual” host‐adapted bacterial colonization, interspecies transmission of antibiotic resistance is associated with specific clonal lineages capable of colonizing both animals and humans. These clones effectively shuttle antibiotic ARGs across host barriers (Gouliouris et al. 2018).

High‐risk antibiotic‐resistant clones are often disseminated through the food chain and the diversity of farm animal populations. For instance, *Staphylococcus aureus* CC97 exemplifies zoonotic transmission from livestock to humans, whereas the MRSA CC398 strain demonstrates reverse transmission from humans to livestock, facilitated by intense selection pressures (Aires‐de‐Sousa 2017). In *Campylobacter jejuni*, the use of fluoroquinolones like enrofloxacin in poultry selects for resistant strains harboring the T86I mutation in the gyrA gene. These resistant strains are then transmitted to humans through contaminated poultry meat. Since the mutation imposes minimal fitness costs in chickens, resistant strains persist on farms even when fluoroquinolone use is limited (Bukari et al. 2025). Similarly, in Salmonella, the multidrug‐resistant *S. enterica* serotype Typhimurium DT104, which carries the characteristic ACSSuT penta‐resistance (ampicillin, chloramphenicol, streptomycin, sulfonamides, and tetracycline) on the Salmonella Genomic Island 1 (SGI1), has spread worldwide among cattle, pigs, poultry, and humans. Genomic analyzes reveal closely related lineages circulating between cattle and human cases across various U.S. states over the past 20 years (Carroll et al. 2023). These examples illustrate the critical need for integrated surveillance and control strategies encompassing human, animal, and environmental health sectors, as emphasized by the One Health paradigm. More recent investigations have expanded to include additional pathogens of significant public health concern, such as resistant *C. jejuni*, with infections reported in humans, animals, and environmental sources (Chibwe et al. 2022). Likewise, MDR *Salmonella* spp. have been identified across human, animal (Mangat et al. 2019), and environmental reservoirs (Dionisi et al. 2011), further emphasizing the interconnected nature of AMR within the One Health framework.

### 3.2 Fundamental Mechanisms of AMR

Over the past decade, there has been a substantial increase in the number of antibiotics to which bacterial populations have developed resistance. This resistance arises through a variety of molecular mechanisms, as illustrated in Figure 3, including the activation of efflux pumps, alterations in cell wall structure, horizontal gene transfer via antibiotic resistance plasmids, and enzymatic inactivation of antimicrobial agents. Among these, efflux pumps represent a major mechanism of resistance, involving membrane‐associated transport proteins that actively export antimicrobial compounds out of the bacterial cell, thereby lowering intracellular drug concentrations and compromising antibiotic efficacy. Efflux systems are classified into five principal families: the ATP‐binding cassette superfamily, the major facilitator superfamily (MFS), the multidrug and toxic compound extrusion family, the small multidrug resistance family, and the resistance‐nodulation‐cell division (RND) family (Piddock 2006). An example is the AcrAB‐TolC efflux pump in *E. coli*, a member of the RND family, which contributes to MDR by extruding a broad range of antibiotics, including β‐lactams and macrolides (Smith et al. 2024).

**Figure 3:**
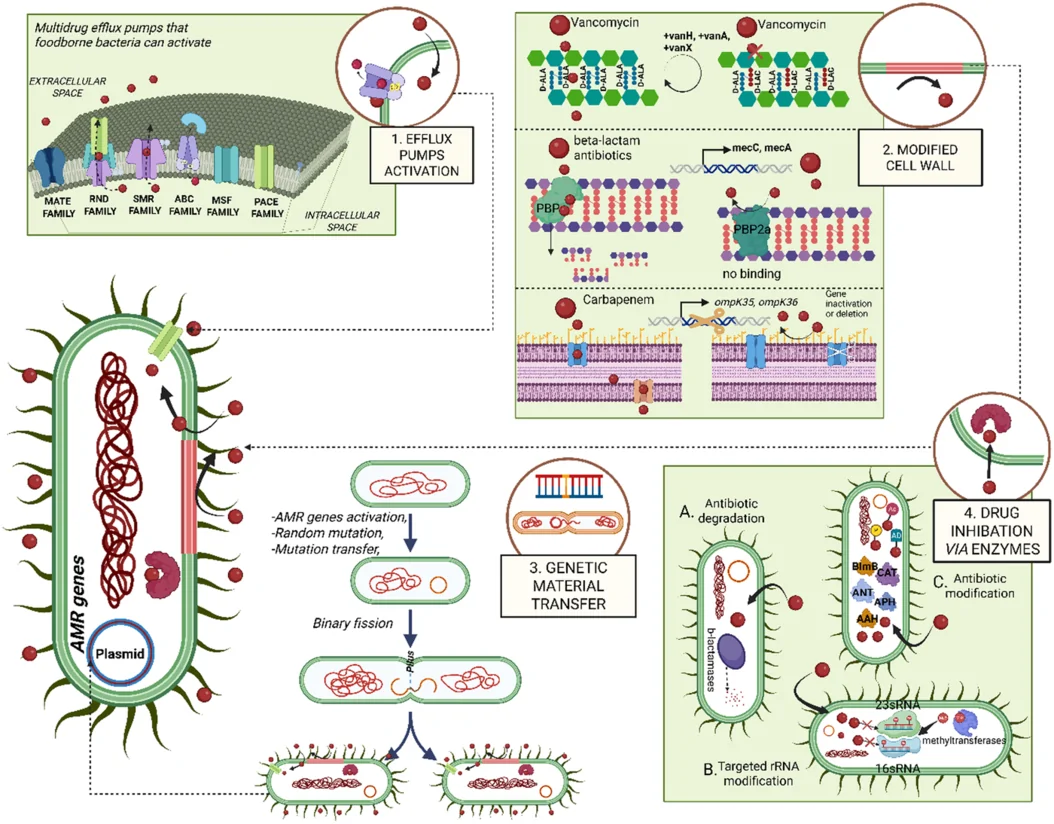
Core genetic and biochemical mechanisms underlying bacterial antimicrobial resistance. (1) Efflux pumps expel antibiotics from the cell; (2) cell wall modifications reduce antibiotic uptake; (3) horizontal gene transfer (HGT) enables sharing of resistance genes; and (4) antibiotic‐inactivating enzymes degrade or modify drugs. These mechanisms collectively sustain the persistence and spread of AMR within food systems.

Modification of the bacterial cell wall is another critical mechanism by which bacteria can acquire resistance to antibiotics. Glycopeptide antibiotics, such as vancomycin and teicoplanin, exert their antibacterial effect by binding to the D‐Ala‐D‐Ala terminus of peptidoglycan precursors, thereby inhibiting the transpeptidation and transglycosylation steps essential for cell wall biosynthesis. Resistance to glycopeptides arises through alteration of the target site, in which the terminal D‐Ala‐D‐Ala is substituted with D‐Ala‐D‐Lactate or D‐Ala‐D‐Serine (Binda et al. 2014). These structural modifications lead to a significant reduction in antibiotic binding affinity (~1000‐fold for D‐Ala‐D‐Lac and ~6‐fold for D‐Ala‐D‐Ser), thereby impairing the antimicrobial action of glycopeptides. The genetic determinants responsible for this resistance were initially identified in clinical isolates, with the *vanA* operon (vanHAX), typically located on the transposon Tn1546, being the most prevalent and clinically relevant genetic cluster associated with high‐level vancomycin resistance (Billot‐Klein et al. 1994).

Enzymatic inactivation of antibiotics represents another major mechanism by which bacteria evade the effects of antimicrobial agents, functioning as an intracellular biochemical shield. Among the most studied examples are the aminoglycoside‐modifying enzymes, which include N‐acetyltransferases (AACs), O‐phosphotransferases (APHs), and O‐adenyltransferases (ANTs). These enzymes inactivate aminoglycoside antibiotics through acetylation, phosphorylation, or adenylylation, respectively, thereby impairing the drug's ability to bind to its ribosomal target. Such self‐protective strategies are not limited to aminoglycosides; for instance, members of the bleomycin antibiotic family (bleomycin, tallysomycin, phleomycin, and zorbamycin) are rendered inactive *via* enzymatic acetylation. In contrast, resistance to β‐lactam antibiotics typically involves hydrolysis of the β‐lactam ring by β‐lactamase enzymes, leading to the loss of bactericidal activity (Peterson and Kaur 2018). Another significant resistance mechanism involves target site modification, particularly among macrolide‐lincosamide‐streptogramin (MLS) antibiotics, which act by binding to the 50S subunit of the bacterial ribosome. Resistance in this context is mediated by methylation of the 23S rRNA at nucleotide position A2058, catalyzed by 23S rRNA methyltransferases. Monomethylation (MLS type I) confers a moderate level of resistance, whereas dimethylation (MLS type II) is associated with high‐level resistance (Douthwaite et al. 2004). Also, resistance to aminoglycosides can be self‐acquired *via* target modification, mediated by 16S rRNA methyltransferases, which catalyze the methylation of specific nucleotides (A1408 or G1405) within the 16S rRNA (Shakil et al. 2008).

### 3.3 Antibiotic Persistence in the One Health Context

Antibiotic persistence, also referred to as heterotolerance, is defined as the ability of a phenotypically distinct subpopulation of bacteria to survive exposure to high concentrations of bactericidal antibiotics without any change in the minimum inhibitory concentration (MIC). A hallmark of this phenomenon is the biphasic killing curve, characterized by rapid elimination of the susceptible majority followed by the prolonged survival of persister cells. Unlike resistance and heteroresistance, which involve heritable or transient increases in MIC, persisters remain non‐growing and metabolically quiescent in the presence of antibiotics. Although the terms persistence and tolerance are often used interchangeably, tolerance refers to reduced killing across the entire population, whereas persistence is a trait exhibited by a subpopulation. Hence, persistence represents a specific form of tolerance marked by population heterogeneity and delayed bacterial clearance (Huemer et al. 2020). Persister cell formation occurs via two principal mechanisms: stochastic induction under normal growth conditions or as a response to environmental stressors (Balaban et al. 2019). The former, historically termed type II persistence (Balaban et al. 2004), involves the spontaneous differentiation of a small subpopulation (typically 0.001%–1%) into a dormant, antibiotic‐tolerant state within an otherwise proliferating isogenic population. Conversely, type I or triggered persistence arises in response to adverse environmental stimuli. Conditions such as nutrient deprivation, antibiotic stress, acidic pH, high cell density, thermal shock, and oxidative stress have been demonstrated to significantly enhance the frequency of persister cell formation, reflecting the adaptive advantage of phenotypic heterogeneity in ensuring survival under hostile conditions.

In the broader context of the One Health framework, antibiotic persistence is increasingly recognized as a critical factor in the long‐term survival and dissemination of bacterial pathogens across ecosystems. In zoonotic and foodborne bacteria, persister cells may contribute to chronic colonization, relapse infections, and horizontal gene transfer, especially in environments where antibiotic residues persist, such as animal production systems and contaminated water sources. The ability of persister cells to survive in diverse ecological niches, including the gut microbiota, soil, and biofilms on medical and agricultural surfaces, poses a significant risk for antimicrobial therapy failure and pathogen resurgence (Bakkeren et al. 2020; Cabral et al. 2018).

## 4 Public Health and Control Strategies

The consumption of animal products, including meat, dairy, and eggs, alongside vegetable products such as fruits and vegetables, is rising due to rapid population growth, urbanization, increased per capita income, globalization, and shifts in consumption patterns. The risk of spreading emerging and re‐emerging antibiotic resistance is heightened by interactions among humans, animals, and ecosystems, resulting in annual fatalities and economic losses (Asfaw et al. 2022).

Effective AMR policies are essential for safeguarding public health, reducing healthcare‐associated infections, and maintaining the efficacy of existing antibiotics. Public health agencies, such as the Centers for Disease Control and Prevention (CDC), the FDA, and the WHO, play a central role in monitoring and addressing AMR. Their efforts include the surveillance of AMR trends, the development of evidence‐based guidelines to reduce antibiotic use in agriculture, and the promotion of research on the transmission pathways of resistant microorganisms. These organizations are also instrumental in designing and implementing risk assessment frameworks that enable the monitoring of AMR throughout the food supply chain, thereby supporting the application of effective prevention and control measures. The WHO classifies several antibiotics used for growth promotion in pigs, poultry, and cattle as critically important for human medicine. However, prolonged exposure to these drugs can drive the emergence of genetic mutations and ARGs, leading to the evolution of resistant bacteria and pathogens (Oliveira et al. 2020).

In the European Union, AMR surveillance in zoonotic and commensal bacteria from food‐producing animals and related food products is conducted annually in a harmonized and legally mandated manner under EU legislation (Directive 2003/99/EC; Decisions 2013/652/EU and 2020/1729). Member States are required to monitor *Salmonella* spp., *C. jejuni*, and *E. coli* isolated from key animal populations and their meat, with sampling alternating between poultry (e.g., broilers, laying hens, turkeys) in even years and pigs or young cattle in odd years. In addition, specific monitoring targets extended‐spectrum beta‐lactamase, AmpC, and carbapenemase‐producing *Salmonella* and *E. coli*. Some countries also include data on methicillin‐resistant *S. aureus*. Surveillance findings, including isolates from human cases of salmonellosis and campylobacteriosis, are compiled and published annually by EFSA and ECDC in the European Summary Reports, providing an integrated view of AMR trends across humans, animals, and food sources (EFSA 2021). The persistent outbreaks and rising incidence of multidrug‐resistant strains highlight the necessity for coordinated surveillance and regulatory measures at both national and international levels.

The WHO and its partners promote the adoption of a One Health approach to effectively tackle the multifaceted and interrelated issues of AMR. This commitment is reflected in the four‐way partnership between FAO, UNEP, WHO, and WOAH, established in 2022 with the One Health Joint Plan of Action (WHO 2022–2026). This plan includes a dedicated action track focused on combating AMR as a global health threat (WHO 2022). The One Health approach tackles AMR through the integration of human, animal, and environmental health strategies aimed at minimizing antimicrobial misuse across various sectors. Coordinated surveillance, responsible antimicrobial use, and cross‐sectoral collaboration, exemplified by initiatives like the Danish DANMAP program, facilitate the tracking of resistance patterns from agricultural settings to clinical environments. This approach recognizes that resistant pathogens may develop in animals, disseminate via food systems or the environment, and ultimately affect human health, complicating treatment of infections and heightening public health risks. One Health promotes systemic responses that preserve the efficacy of current antimicrobials while protecting the interconnected health of humans, animals, and ecosystems (WHO 2024a).

This integrative framework is enhanced by global surveillance systems, such as the WHO's GLASS initiative, which operationalizes the One Health approach through the standardization of AMR data collection across human, animal, and environmental domains. GLASS, launched by the WHO in 2015, provides a standardized framework for global AMR data collection and reporting. It supports the One Health approach by integrating human, animal, and environmental data, monitoring resistance in key bacterial pathogens and antimicrobial use. Despite implementation challenges in low‐resource settings, GLASS has improved global AMR surveillance through capacity building, digital tools, and international collaboration (Majumder et al. 2020; Seale et al. 2017; Serwecińska 2020).

Global increase in outbreaks of bacterial gastrointestinal infections places resistant foodborne pathogens at a critical point on the food safety map. Neither pathogenic bacteria nor antibiotics are naturally present in vegetables and other raw foods. The discovery of bacteria as a cause of disease and antibiotics as effective treatments has significantly improved human and animal health over the past century. However, the global rise of AMR, driven by factors such as excessive antibiotic use in human medicine, livestock, and agriculture, as well as environmental contamination, now threatens the efficacy of these life‐saving drugs. AMR is a complex, multifactorial issue that involves both selection (via antibiotic pressure) and transmission (across the human–animal–environment interface), requiring a coordinated, multidisciplinary One Health approach to surveillance, prevention, and control.

Mitigating AMR in the food chain extends beyond surveillance and governance, requiring practical measures that decrease antimicrobial use at the source. On farms, improvements in husbandry practices, stronger biosecurity, and vaccination programs help lower disease prevalence and consequently reduce the need for therapeutic and prophylactic antibiotics. Vaccines against pathogens like Salmonella and avian pathogenic *E. coli* have demonstrated notable success in cutting antibiotic usage in livestock (Hoelzer et al. 2018). Simultaneously, a variety of non‐antibiotic options (probiotics, prebiotics, organic acids, phytogenic compounds, antimicrobial peptides, and bacteriophages) are increasingly adopted to support animal health and productivity following the restrictions on growth promoters (Helmy et al. 2023). Further along the supply chain, rigorous slaughtering and processing procedures, strict adherence to HACCP principles, and effective post‐harvest sanitization measures help minimize the survival of resistant bacteria and cross‐contamination before products reach consumers.

By promoting responsible antibiotic use, advancing surveillance, and encouraging innovation in antimicrobial development, these policies contribute to the sustainability and resilience of healthcare systems worldwide. Anchored in international frameworks such as the WHO Global Action Plan and the EU's One Health strategy, global and national action plans (ranging from the U.S. and Australia to China, India, and South Africa) reflect a unified multisectoral commitment to mitigating AMR through coordinated governance, research, and stewardship.

Consumers play a crucial role in the last line of defense, as foods prepared at home significantly contribute to foodborne illnesses. The WHO advocates for the “Five Keys to Safer Food”: a set of practical guidelines shared worldwide: keep clean (wash hands and surfaces), separate raw and cooked foods to prevent cross‐contamination, cook foods thoroughly, maintain safe temperatures (below 5°C or above 60°C), and use safe water and raw ingredients (WHO 2006). Variations like the “Five Keys to Growing Safer Fruits and Vegetables” and the “Five Keys to Safer Aquaculture Products” expand these principles to primary production. Since resistant bacteria are most often spread in kitchens via unwashed hands, knives, and cutting boards, consistently following these hygiene practices provides an effective barrier against the spread of AMR bacteria to humans (Mataragka et al. 2025).

## 5 Conclusion

AMR is a complex, multifactorial threat that extends beyond clinical settings and into the core of the food systems. ARB and ARGs are disseminated through multiple pathways, ranging from intensive animal farming and aquaculture to plant agriculture and post‐harvest food handling, making food a significant but often overlooked vehicle for AMR transmission. The widespread and, in many cases, unregulated use of antibiotics for growth promotion, prophylaxis, and metaphylaxis in livestock and aquaculture has led to the selection of resistant bacterial populations that persist throughout the food production chain. Moreover, environmental vectors such as irrigation water, contaminated soil, and manure‐based fertilizers further contribute to the contamination of plant‐based foods, compounding the risk of exposure to resistant organisms.

Bacteria such as *E. coli*, *K. pneumoniae, S. aureus*, and *A. baumannii*, which are listed by the WHO as critical‐priority pathogens, have been repeatedly isolated from animal‐derived and plant‐origin food products. The persistence of these microorganisms is facilitated by a variety of resistance mechanisms, including efflux pumps, enzymatic degradation of antimicrobials, biofilm formation, and horizontal gene transfer. These processes are not constrained to individual hosts or environments; rather, they represent a continuum across ecological compartments, human, animal, and environmental, highlighting the intricate and interdependent nature of AMR dynamics in food systems. Yet, despite accumulating evidence, the role of the agri‐food sector in the propagation of AMR remains insufficiently recognized in both research and policy arenas. Current interventions often focus predominantly on clinical settings, overlooking the substantial selective pressures exerted within food production environments. Surveillance systems are fragmented, regulatory frameworks are inconsistent across countries, and data on non‐clinical AMR reservoirs remain sparse, particularly in low‐ and middle‐income regions where antibiotic use is poorly controlled.

A paradigm shift is urgently needed, one that embraces the One Health framework as a foundational principle for action. Tackling AMR in food systems requires integrative, cross‐disciplinary strategies that bridge the gaps between human medicine, veterinary science, plant pathology, environmental health, and food safety. Such strategies must include: (i) the development and enforcement of harmonized international standards for antibiotic use in agriculture; (ii) investment in robust surveillance systems capable of tracking AMR trends across the entire farm‐to‐fork continuum; (iii) promotion of good agricultural practices, including alternatives to antibiotic use such as probiotics, vaccines, and improved biosecurity; and (iv) public education campaigns to raise awareness about AMR risks associated with food consumption. Failure to act decisively will allow AMR to further entrench itself as a global food safety and health crisis. Conversely, coordinated and sustained efforts across sectors offer a path forward, one that not only mitigates AMR but also enhances the resilience and sustainability of food systems worldwide. Recognizing and addressing antibiotic resistance in food is no longer optional; it is a scientific and moral imperative in the broader fight against one of the defining public health challenges of our time.

## Author Contributions

**Răzvan Odocheanu:** writing – original draft, writing – review and editing, formal analysis, methodology, software, data curation. **Madalina Lorena Medeleanu:** writing – original draft, methodology, writing – review and editing, software, formal analysis, data curation. **Doru Pamfil:** validation, writing – review and editing, visualization. **Dan Cristian Vodnar:** conceptualization, visualization, validation, writing – review and editing, project administration, supervision.

## Funding

This work was supported by the Romanian Ministry of Research, Innovation and Digitalisation grant number 17 PCE/2025, project no. PN‐IV‐P1‐PCE‐2023‐1092.

## Ethics Statement

The authors have nothing to report.

## Conflicts of Interest

The authors declare no conflicts of interest.

## Data Availability

Data sharing not applicable to this article as no datasets were generated or analyzed during the current study.
